# Influence of Step Frequency on the Dynamic Characteristics of Ventilation and Gas Exchange During Sinusoidal Walking in humans

**DOI:** 10.3389/fphys.2022.820666

**Published:** 2022-04-12

**Authors:** Mako Fujita, Kiyotaka Kamibayashi, Tomoko Aoki, Masahiro Horiuchi, Yoshiyuki Fukuoka

**Affiliations:** ^1^Faculty of Health and Sports Science, Doshisha University, Kyoto, Japan; ^2^Faculty of Environmental Symbiotic Science, Prefectural University of Kumamoto, Kumamoto, Japan; ^3^Division of Human Environmental Science, Mt. Fuji Research Institute, Fujiyoshida, Japan

**Keywords:** ventilation, breath frequency, step frequency, stride length, entrainment

## Abstract

We tested the hypothesis that restricting either step frequency (SF) or stride length (SL) causes a decrease in ventilatory response with limited breath frequency during sinusoidal walking. In this study, 13 healthy male and female volunteers (mean ± SD; age: 21.5 ± 1.8 years, height: 168 ± 7 cm, weight: 61.5 ± 8.3 kg) participated. The walking speed was sinusoidally changed between 50 and 100 m⋅min^–1^ with periods from 10 to 1 min. Using a customized sound system, we fixed the SF at 120 steps⋅min^–1^ with SL variation (0.83–0.41 m) (*SF*_*fix*_) or fixed the SL at 0.7 m with SF variation (143–71 steps⋅min^–1^) (*SL*_*fix*_) during the subjects’ sinusoidal walking. Both the subjects’ preferred locomotion pattern without a sound system (*Free*) and the unprompted spontaneous locomotor pattern for each subject (*Free*) served as the control condition. We measured *breath-by-breath* ventilation [tidal volume (VT) and breathing frequency (B*f*)] and gas exchange [CO_2_ output ($\dot{\text{V}}$CO_2_), O_2_ uptake ($\dot{\text{V}}$O_2_)]. The amplitude (*Amp*) and the phase shift (PS) of the fundamental component of the ventilatory and gas exchange variables were calculated. The results revealed that the *SF*_*fix*_ condition decreased the *Amp* of the B*f* response compared with *SL*_*fix*_ and *Free* conditions. Notably, the *Amp* of the B*f* response under *SF*_*fix*_ was reduced by less than one breath at the periods of 5 and 10 min. In contrast, the *SL*_*fix*_ condition resulted in larger Amps of B*f* and $\dot{\text{V}}$_*E*_ responses as well as *Free*. We thus speculate that the steeper slope of the $\dot{\text{V}}$_*E*_-$\dot{\text{V}}$CO_2_ relationship observed under the *SL*_*fix*_ might be attributable to the central feed-forward command or upward information from afferent neural activity by sinusoidal locomotive cadence. The PSs of the $\dot{\text{V}}$_*E*_, $\dot{\text{V}}$O_2_, and $\dot{\text{V}}$CO_2_ responses were unaffected by any locomotion patterns. Such a sinusoidal wave manipulation of locomotion variables may offer new insights into the dynamics of exercise hyperpnea.

## Introduction

Humans’ sinusoidal exercising is clearly voluntary rhythmic movement in response to the stress of a varying work rate or speed. The resulting exercise-induced hyperpnea is expected to be integrated with chemical feedback from both central (Bell, 2006) and peripheral chemoreceptors (Forster et al., 2012; Ebine et al., 2018), afferent neural activity from working muscles (Eldridge et al., 1982; Eldridge, 1994; Amann et al., 2010; Forster et al., 2012), and feedforward signals from the motor cortex (Comroe and Schmidt, 1943; Dejours, 1959; Eldridge et al., 1981).

Several previous studies have used sinusoidal work forcing to define the resulting kinetics of cardiac, ventilatory, and gas exchange responses (Casaburi et al., 1977, 1978; Fukuoka et al., 2017; Ebine et al., 2018), and it is well established that these responses to a sinusoidal workload will have a consistent fundamental frequency component, which can be characterized by a mean value (*Mx*), an amplitude (*Amp*), and a phase shift (*PS*) on gas exchange kinetics (Casaburi et al., 1977). Moreover, several investigators have observed that for the same increase in metabolic rate, the hyperpnea is greater when the treadmill speed or pedaling frequency during cycling is increased, as opposed to an increase in the treadmill grade or cycling resistance (Bechbache and Duffin, 1977; Bramble and Carrier, 1983; Casey et al., 1987; Lafortuna et al., 1996). During sinusoidal exercise, greater Amps and lower PSs for pulmonary ventilation ($\dot{\text{V}}$_*E*_) were observed when the limb-movement frequency was varied sinusoidally by alterations in cycling work rates (Casaburi et al., 1977) or in treadmill speed (Wells et al., 2007). The PS of $\dot{\text{V}}$_*E*_ dynamics during a sinusoidal leg-cycling exercise was less for the cadence variation than for the sinusoidal pedal forcing variation (Duffin, 2014). It may also be possible that the sinusoidal step frequency (SF) of walking accelerates the ventilatory dynamics as the SF is equivalent to pedaling cadence.

Sinusoidal exercise protocols can be considered a robust method to investigate multiple factors that affect the ventilatory response (Caterini et al., 2016), and these protocols may be suitable for avoiding the entrainment of breath frequency to limb movement (Bechbache and Duffin, 1977). In this study, the SF was forced sinusoidally to minimize contributions from locomotor-respiratory entrainment even at a constrained stride length (SL) during sinusoidal walking (Caterini et al., 2016). The variation in the SF would induce sufficient exercise hyperpnea, possibly involving muscle reflex drives from type III and IV afferent neural activities (Bruce et al., 2019; Ward, 2019).

The synchronization of limb movement and breathing rhythms has been observed in locomoting animals (Bechbache and Duffin, 1977; Bramble and Carrier, 1983; Lafortuna et al., 1996; Boggs, 2002) as locomotor-respiratory entrainment. A limb movement-related afferent signal can entrain the ongoing respiratory rhythm (Giraudin et al., 2012). We thus speculated that during sinusoidal walking, a constrained SF could reinforce locomotor-respiratory entrainment and consequentially the breath frequency (B*f*) would remain constant.

Accordingly, we addressed two specific aims in this study: (i) the locomotive cadence (i.e., the SF) may be a significant factor to cause exercise-induced hyperpnea, possibly involving muscle reflex drives from type III and IV afferents (Haouzi et al., 2004; Haouzi, 2006; Bruce et al., 2019) or the central feed-forward command (i.e., feedforward mechanism from the higher central nervous system to locomotor and respiratory neurons) (Eldridge and Waldrop, 1991; Waldrop et al., 1996; Bell, 2006); and (ii) locomotion with a fixed SF may cause dynamic ventilatory depression (i.e., Amp depression in ventilation) as a secondary result of constrained B*f* due to a respiratory-locomotor network (Le Gal et al., 2020). To test this hypothesis, we investigated whether the ventilatory and gas exchange responses showed different dynamics in conditions with different locomotion patterns during walking with sinusoidal speed changes.

## Materials and Methods

### Subjects

The subjects were 13 healthy young male (*n* = 7) and female (*n* = 6) volunteers [age: 21.5 ± 1.8 years, height: 168 ± 7 cm, weight: 61.5 ± 8.3 kg; mean ± standard deviation (SD)] who were not taking any medication that could affect cardiovascular responses. The subjects were fully informed of any risks and discomforts associated with these experiments before giving their written, informed consent to participate in the study, which was approved by the ethics committees of the Institutional Review Board of Doshisha University (no. 1045).

### Protocols

**FIGURE 1:**
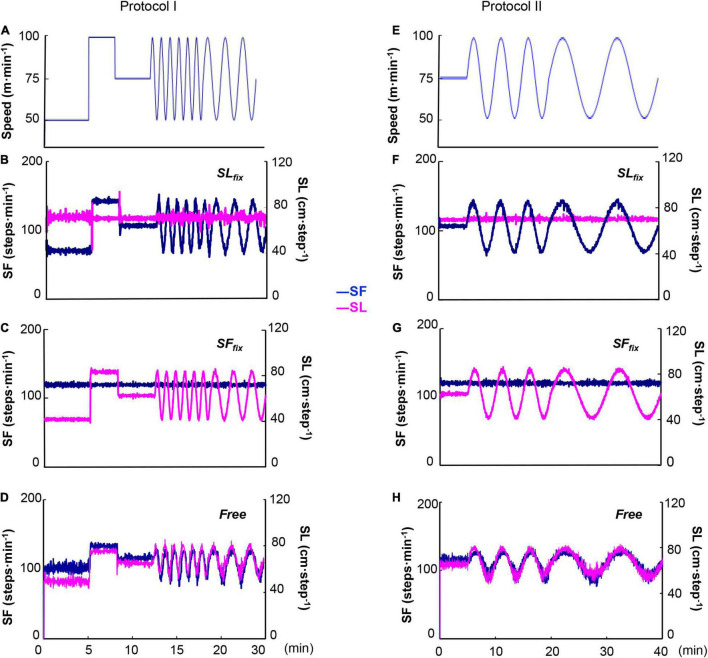
Two sinusoidal locomotion protocols at a sinusoidal speed between 50 and 100 m⋅min^–1^ at the period of 1⋅2 min **(A)** and 5⋅10 min **(E)**. The stride length (SL) was fixed at 0.7 m with step frequency (SF) variation (143–71 step⋅min^–1^) (*SL*_*fix*_) **(B,F)** and the SF was fixed at 120 steps⋅min^–1^ with stride variation (0.83–0.41 m) (*SF*_*fix*_) **(C,G)**. The control (*Free*) condition was the subjects’ preferred locomotion pattern **(D,H)**.

All experimental tests were completed in a temperature-controlled laboratory (25 ± 0.4°C with 50 ± 3% relative humidity), and all subjects wore underwear, shorts, and a T-shirt, as well as socks and shoes. The protocols used herein are based on our previous work (Fukuoka et al., 2017). The treadmill speed was changed in a sinusoidal pattern from 50 to 100 m⋅min^–1^ at periods (T) of 10, 5, 2, and 1 min. A warm-up session consisted of steady-state walking for 4 or 6 min, which preceded each recording sinusoidal exercise session. In Protocol I, after 50, 100, and 75 m⋅min^–1^ for 5, 3, and 4 min of warm-up walking, the sinusoidal loading was repeated for five cycles at 1-min periods, followed by three cycles at 2-min periods (Figure 1A). In Protocol II, after 75 m⋅min^–1^ for 6 min of warm-up walking, the sinusoidal loading was repeated for three cycles at 5-min periods, followed by two cycles at 10-min periods (Figure 1E). A microcomputer transmitted the signal controlling the speed of the motor driving the treadmill (modified TMS 2200, Nihon Koden, Tokyo) through a digital-analog converter.

Using a customized personal computer sound cueing system (Arco Metronome, Ver.2.00, Arco System, Chiba, Japan), we set two locomotion patterns. In the fixed SL (*SL*_*fix*_) condition, the SL was fixed at 0.7 m and the SF varied between 143 and 71 steps⋅min^–1^ in coordination with the sinusoidal changes in treadmill speed (Figures 1B,F; Latt et al., 2008). In the fixed SF (*SF*_*fix*_) condition, the SF was set at 120 steps⋅min^–1^ with SL variation (0.83–0.41 m) during sinusoidal walking (Figures 1C,G; Casaburi et al., 1978). The subjects were instructed to synchronize their locomotion to the sound. In addition, the subjects were allowed to breathe freely, so breathing and walking were not intentionally synchronized. The subject’s preferred locomotion pattern without the sound was used as the control condition (*Free*) (Figures 1D,H). The subjects performed each protocol on six separate occasions metricconverterProductID0.7 m(one session at a time, three sessions per week for each individual).

### Measurements

A mass-flow sensor (type AB, Minato Medical Sciences, Osaka) was fit to the expiratory port of the valve of the face mask worn by the subject to continuously record the subject’s expiratory airflow, which was calibrated before each measurement with a 3-L syringe at three different flow rates. We calculated the ventilation ($\dot{\text{V}}$_*E*_) values by integrating the tidal volume (VT; L) and breathing frequency (B*f*; breaths⋅min^–1^). The end-tidal oxygen pressure (P_*ET*_O_2_; mmHg) and carbon dioxide pressure (P_*ET*_CO_2_; mmHg) were determined using mass spectrometry (Arco-2000, Arco System, Chiba, Japan) from a sample drawn continuously from the inside of the face mask at 1 ml⋅s^–1^. This loss of gas volume was not examined in this study, because the loss of 1 ml⋅s^–1^ was much smaller than the inspired and expired airflows. Three reference gases of known concentrations (O_2_: 15.04%, CO_2_: 2.92%, and N_2_: 82.04%; O_2_: 11.93%, CO_2_: 6.96%, and N_2_: 81.11%) and room air (O_2_: 20.93%, CO_2_: 0.03%, Ar: 0.94%, and N_2_: 78.10%) were used to calibrate the mass spectrometer.

The volumes, flows, partial pressure of carbon dioxide (PCO_2_), and partial pressure of oxygen (PO_2_) at the subject’s mouth were recorded in real time with a 50-Hz sampling frequency using a computerized online breath-by-breath system (AE-280, Minato Medical Sciences, Osaka) from the time-aligned gas volume and concentration signals. Breath-by-breath $\dot{\text{V}}$_*E*_ (BTPS), $\dot{\text{V}}$O_2_ (STPD), and $\dot{\text{V}}$CO_2_ (STPD), VT, B*f*, P_*ET*_CO_2_, and P_*ET*_O_2_ were determined. An electrocardiogram (ECG) was recorded using a bioamplifier (AB 621G, Nihon Kohden, Tokyo). Heart rate (HR) was measured by beat-by-beat counting from the *R* spike of the ECG. The signals from the treadmill were fed into a data acquisition system (PowerLab system, A/D Instruments, Castle Hill, NSW, Australia) and temporally aligned to the ventilatory and ECG data.

In all subjects, the SF and SL were measured using a switch activated by the subject stepping on a sensor on the sole of the right foot of the right leg in each protocol. The signals from the treadmill and the stepping sensor were fed into the PowerLab data acquisition system and temporally aligned with the ventilatory data.

### Data Analysis

All the cardiorespiratory and locomotive data were analyzed using a Fourier analysis as previously reported (Wigertz, 1971; Haouzi et al., 1992, 2004; Fukuoka et al., 2017). The breath-by-breath ventilatory and gas exchange data were interpolated into a 1-s interval value before Fourier analysis (Supplementary Figure 1). The repeated cardiorespiratory responses to sinusoidal walking speed were superimposed in correspondence with the cycle period, and we obtained the average cardiorespiratory data at each respective cycle. The variation in the speed of the treadmill was regarded as the input function. The *Amp* (i.e., mean to peak) and the *PS* of the fundamental component (the same frequency as the input function) of the $\dot{\text{V}}$_*E*_, $\dot{\text{V}}$O_2_, $\dot{\text{V}}$CO_2_, HR, and end-tidal PCO_2_ (P_*ET*_CO_2_) responses as well as the locomotion responses (locomotion SF and SL) were computed as follows:


(1) $$A\,m\,p=\sqrt{R\,e^{2}+I\,m^{2}}$$

and


(2) PS=tan(R⁢eI⁢m)-1

where the *R*e and *I*m are the real and imaginary components; these were calculated as follows. The larger the *PS*, the slower the response. The larger the *Amp*, the higher the responsiveness.


(3) $$R\,e=\frac{2}{N\,T}\,\sum_{t=0}^{N\,T}\left[\left(x\,\left(t\right)-M\,x\right)\,\cos\,\left(2\,\pi \,f\,\text{t}\right)\right]$$

and


(4) $$I\,m=\frac{2}{N\,T}\,\sum_{t=0}^{N\,T}\left[\left(x\,\left(t\right)-M\,x\right)\,\sin\,\left(2\,\pi \,f\,\text{t}\right)\right]$$

where *x*(*t*) is the response value at time *t* (in s), *M*x is the mean value of *x* for an integer number of cycles (*N*), *T* is the period of the input signal (in s), and *f* ( = 1/*T*) is its frequency in cycles per second.

We normalized the ratios of *Amp* of the respiratory and locomotion variables against sinusoidal change in walking speed by dividing the magnitude of variables from 50 to 100 m min^–1^ during each steady-state exercise, and the results are presented as the *Amp* ratio (%) (Fukuoka et al., 2017).

The R-R intervals during sinusoidal work were calculated beat-by-beat by the computer, and 1-s interval HR data were measured from the calculated R-R intervals (R-R) and converted as HR values (60/R-R). The subject’s locomotion SF and SL were measured with a switch activated by stepping on a sensor on the sole of the right foot in each protocol (Fukuoka et al., 2017).

### Statistical Analyses

All values are presented as mean ± SD. The significance of differences in each variable ($\dot{\text{V}}$E, VT, B*f*, $\dot{\text{V}}$O_2_, $\dot{\text{V}}$CO_2_, and HR) was determined by a two-way repeated-measures analysis of variance (ANOVA) in the comparison of the three locomotion patterns (*SF_*fix*_, SL_*fix*_*, and *Free*) × oscillation frequency period (*T*; 1 to 10 min). *Bonferroni’s* test was applied for the appropriate datasets if a significant *F*-value was obtained. We compared the regression coefficients of the independent variables of $\dot{\text{V}}$_*E*_ of the three locomotion patterns (*SF_*fix*_, SL_*fix*_*, and *Free*). The level of significance was set at *p* < 0.05.

## Results

**FIGURE 2:**
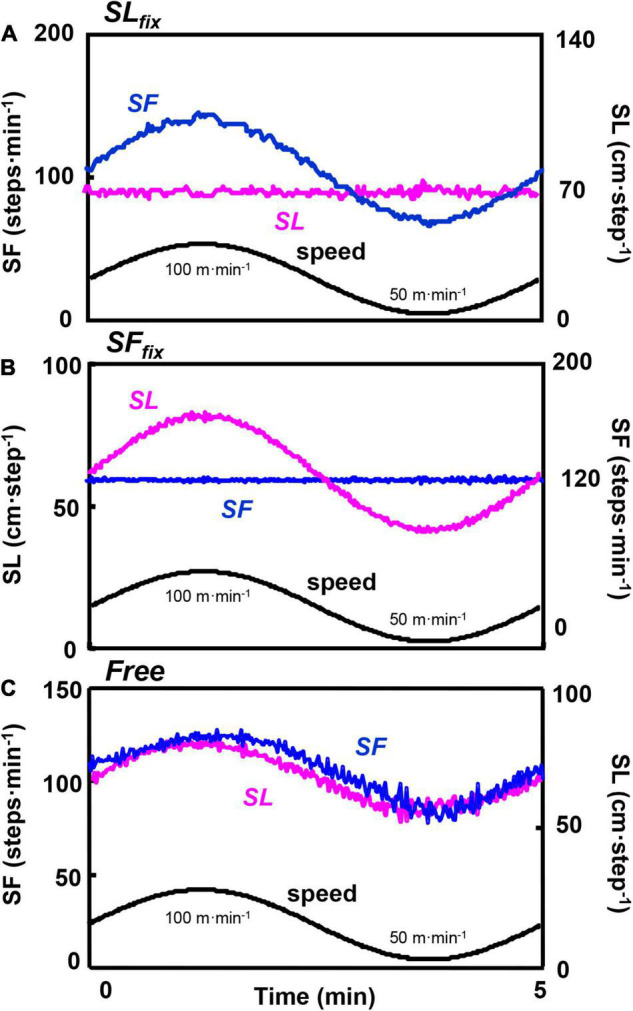
Time course of the locomotion responses at a 5-min period. Example from a representative subject of the locomotion pattern of the step frequency (SF) and the stride length (SL) divided under *SL*_*fix*_ **(A)**, *SF*_*fix*_ **(B)**, and *Free* **(C)**. The *oscillating line* is the superimposed SL and SF for a 5-min period. The *lower sinusoidal curve* is the sinusoidal change of the speed between 50 and 100 m⋅min^–1^.

Figure 2 shows the representative smoothing *SL*_*fix*_, *SF*_*fix*_, and *Free* locomotion patterns at the period of 5 min. The *SL*_*fix*_ and *SF*_*fix*_ were strictly controlled (Figures 2A,B). In *Free* locomotion, both the SL and SF responded almost synchronously with the sinusoidal walking speed (Figure 2C).

**FIGURE 3:**
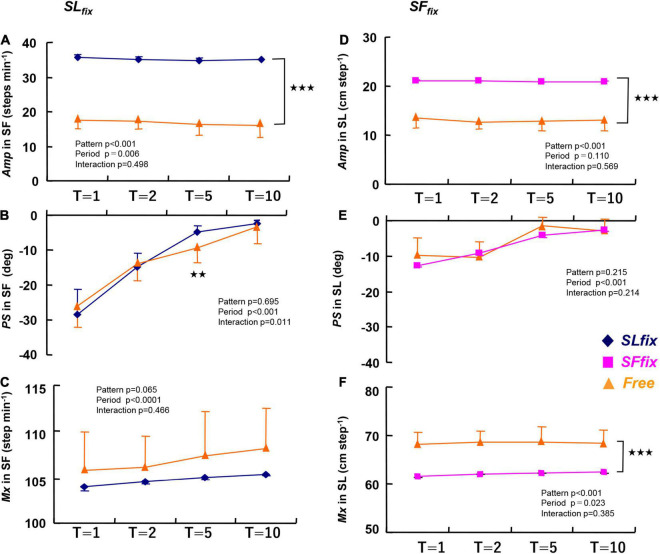
The amplitude (*Amp*), *phase shift (PS)*, and mean (*Mx*) *values* in the step frequency under *SL*_*fix*_ **(A–C)** and in the stride length under *SF*_*fix*_ **(D–F)** compared with *Free*. The *Amp* under both *SL*_*fix*_ and *SF*_*fix*_ became significantly higher compared with those at *Free* at any period (*p* < 0.001). The *PS* values for SL were quite small and virtually close to zero at each period **(E)**. The *Mx* under *SL*_*fix*_ tended to be lower and the *Mx* under *SF*_*fix*_ was significantly lower than that under the *Free* condition at each period. **p < 0.01, ***p < 0.001 vs. *Free*. T, time periods (1, 2, 5, and 10 min). Data are mean ± SD.

The *Amp*, *PS*, and *Mx* under the *SF*_*fix*_ or *SL*_*fix*_ conditions at all periods (T: 1, 2, 5, and 10 min) are given in Figure 3. Compared with the *Free* locomotion, the *Amp* of the *SF* under the *SL*_*fix*_ condition was significantly greater at all periods (pattern effect: *p* < 0.001, η^2^ = 0.983) (Figure 3A). The *M*x value for SF tended to be greater in the *Free* conditions than in the *SL*_*fix*_ condition (pattern effect: *p* = 0.065, η^2^ = 0.255) (Figure 3C). The *PS* for SF was not significantly different between the *Free* and *SL*_*fix*_ conditions except at the 5-min period (*p* < 0.01) (Figure 3B).

The *Amp* of the SL under the *SF*_*fix*_ condition was significantly larger than that under *Free* at all periods (pattern effect: *p* < 0.001, η^2^ = 0.965) (Figure 3D). In contrast, the *Mx* for the SL became significantly lower under *SF*_*fix*_ compared to *Free* (pattern effect: *p* < 0.001, η^2^ = 0.877) (Figure 3F). There were no significant differences in the *PS* for the SL at any period (Figure 3E).

### Dynamic Responses of Ventilatory Variables

**FIGURE 4:**
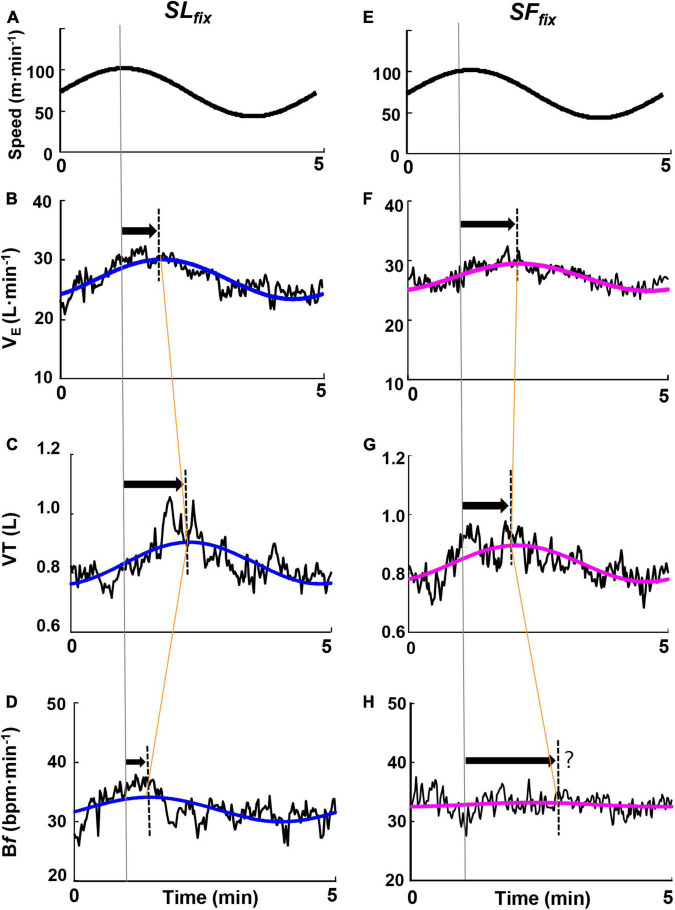
Time course of the ventilatory responses to the sinusoidal change in treadmill speed at the period of 5 min. Examples highlighting characteristic responsiveness of the ventilatory variables of ventilation ($\dot{\text{V}}$_*E*_), breath frequency (*Bf*), and tidal volume (VT) responses under *SL*_*fix*_ locomotion **(A–D)** and *SF*_*fix*_ locomotion **(E–H)** in a representative subject. The *oscillating line* is the superimposed gas exchange variables data. The *smooth blue and red lines* are the sine-wave fundamental component of these dynamics.

Figure 4 illustrates the responses of ventilatory variables at the 5-min period for a representative single same subject, revealing that the VT and B*f* showed markedly different responses. Specifically, under the *SL*_*fix*_ condition, the *PS* for the B*f* response was preceded by a delay in VT (i.e., a late appearance of the peak in VT), whereas under the *SF*_*fix*_ condition, the *PS* for the VT response was preceded by a delay in the B*f* (i.e., a late appearance of the peak of B*f* or an almost constant).

**FIGURE 5:**
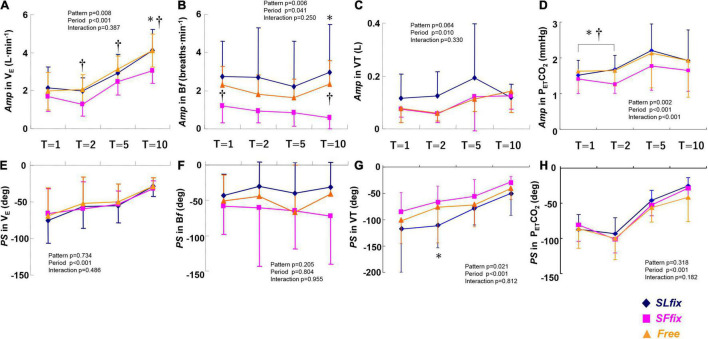
Comparisons of *Amp* and *PS* in $\dot{\text{V}}$_*E*_ **(A,E)**, *Bf* **(B,F)**, VT **(C,G)**, and end-tidal PCO_2_ (P_*ET*_CO_2_) **(D,H)** dynamics under *SL_*fix*_, SF*_*fix*_, and *Free*. *p < 0.05 between *SL*_*fix*_ vs. *SF*_*fix*_ within the same period. ^†^*p* < 0.05 between *Free* vs. *SF*_*fix*_ within the same period. T, time periods (1, 2, 5, and 10 min). Data are mean ± SD.

The *SF*_*fix*_ condition induced significantly lower *Amp*s in $\dot{\text{V}}$_*E*_ and P_*ET*_CO_2_ at allmetricconverterProductID1 A of the periods compared with the *SL*_*fix*_ condition, with similar values between the *SL*_*fix*_ and *Free* conditions (Figures 5A,D). The *Amp* response for the B*f* remained unchanged (<1.0 breath⋅min^–1^) under *SF*_*fix*_ (Figure 5B), with a pattern effect among the three patterns (pattern effect: *p* = 0.006, η^2^ = 0.348). The *Amp* of the VT response tended to be larger under *SL*_*fix*_ compared with *SF*_*fix*_ and Free at all periods (*p* = 0.064, η^2^ = 0.204) (Figure 5C). With respect to the *PS*, a significant main effect of patterns was observed in the VT (pattern effect: *p* = 0.021, η^2^ = 0.275) (Figure 5G). A Bonferroni *post-hoc* test further revealed that the *PS* for the VT at the 2-min period under the *SL*_*fix*_ condition was significantly lower than under the *SF*_*fix*_ condition (*p* < 0.05).

However, we did not observe any effects of the patterns on the *PS* in $\dot{\text{V}}$_*E*_, B*f*, or P_*ET*_CO_2_metricconverterProductID1 C, with period effects for $\dot{\text{V}}$_*E*_ and P_*ET*_CO_2_ (Figures 5E,F,H). In addition, a significant main effect of the pattern was observed on the *Mx* in $\dot{\text{V}}$_*E*_ (pattern effect: *p* = 0.012, η^2^ = 0.309), with a significant difference between the *Free* and *SL*_*fix*_ conditions at only the 5-min period (Supplementary Table 1, *p* = 0.015). There were no significant main effects of the locomotion patterns or frequency period, and no interaction effect in the *Mx* for VT, B*f*, and P_*ET*_CO_2_ (all *p* > 0.05, Supplementary Table 1).

### Dynamic Responses of Gas Exchange and Heart Rate Variables

**FIGURE 6:**
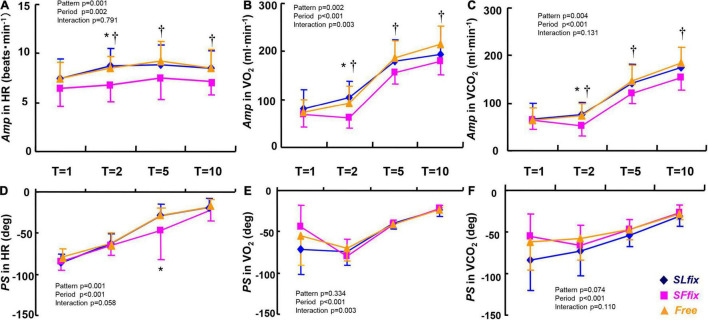
Comparisons of *Amp* and *PS* in heart rate (HR) **(A,D)**, O_2_ uptake ($\dot{\text{V}}$O_2_) **(B,E)**, and CO_2_ output ($\dot{\text{V}}$CO_2_) **(C,F)** dynamics under *SL*_*fix*_, *SF*_*fix*_, and *Free*. *p < 0.05 between *SL*_*fix*_ vs. *SF*_*fix*_ within the same period. ^†^*p* < 0.05 between *Free* vs. *SF*_*fix*_ within the same period. T, time periods (1, 2, 5, and 10 min). Data are shown in mean ± SD.

The *Amp* values of the HR, $\dot{\text{V}}$O_2_, and $\dot{\text{V}}$CO_2_ variables were significantly lower under *SF*_*fix*_ compared with *SL*_*fix*_ and *Free* (pattern effect: *p* = 0.001, 0.002, and 0.004, and η^2^ = 0.446, 0.408, and 0.370, respectively, Figures 6A–C). Although the period had significant main effects on the *PS* of HR, $\dot{\text{V}}$O_2_, and $\dot{\text{V}}$CO_2_ (Figures 6D–F), those values were similar irrespective of the locomotion patterns, with the exception of a greater *PS* in the HR at 5 min under *SF*_*fix*_. We also observed no significant main effects of the locomotion patterns and frequency period, and no interaction effect in the *Mx* of $\dot{\text{V}}$CO_2_, $\dot{\text{V}}$O_2_, and HR (all *p* > 0.05, Supplementary Table 2).

### Relationship Between the Amp Ratio in $\dot{\text{V}}$_*E*_ and $\dot{\text{V}}$CO_2_

**FIGURE 7:**
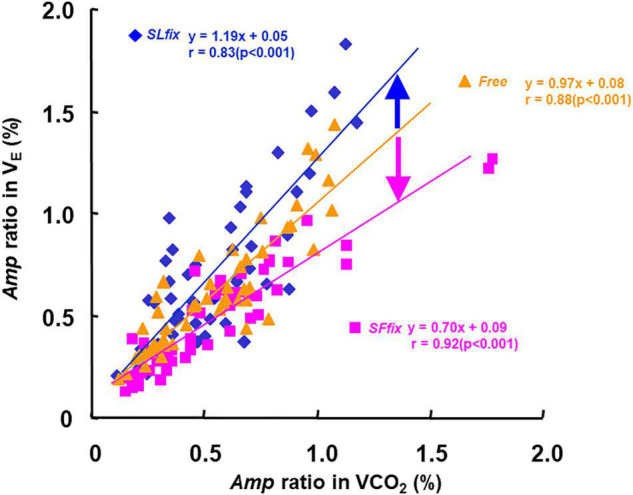
Relationship between the *Amp* ratio in $\dot{\text{V}}$_*E*_ and $\dot{\text{V}}$CO_2_ under *SL*_*fix*_, *SF*_*fix*_, and *Free*, respectively. The slope of the regression lines of the $\dot{\text{V}}$_*E*_-$\dot{\text{V}}$CO_2_ relationship was steeper at *SL*_*fix*_ than *SF*_*fix*_. *SL*_*fix*_: *y* = 1.19x + 0.05, *r* = 0.83 (*p* < 0.001). *SL*_*fix*_: *y* = 0.70x + 0.09, *r* = 0.92 (*p* < 0.001). *Free*: *y* = 0.97x + 0.08, *r* = 0.88 (*p* < 0.001).

The *Amp* ratio in the $\dot{\text{V}}$_*E*_ (i.e., *Amp* ratio of sinusoidal forcing variations to constant) was closely related to the *Amp* ratio in the $\dot{\text{V}}$CO_2_ when the data from all periods were pooled (*SL*_*fix*_: *r* = 0.83, *SF*_*fix*_: *r* = 0.88, and *Free*: *r* = 0.91, *p* < 0.01) (Figure 7). The slope of the regression lines of the $\dot{\text{V}}$_*E*_−$\dot{\text{V}}$CO_2_ relationship was steeper under *SL*_*fix*_ (s: 1.19) than under *SF*_*fix*_ (s: 0.70) and *Free* conditions (s: 0.97).

## Discussion

The three major findings of this study are as follows: (i) the *SL*_*fix*_ locomotion pattern increased the *Amp* of $\dot{\text{V}}$_*E*_ (Figure 5A) and metabolic responses ($\dot{\text{V}}$O_2_ and $\dot{\text{V}}$CO_2_; Figures 6B,C) compared with the *SF*_*fix*_ (i.e., 120 steps⋅min^–1^); (ii) the *Amp* of the B*f* under the *SF*_*fix*_ locomotion pattern remained unchanged (i.e., <1.0 breath⋅min^–1^); and (iii) when the slope of the $\dot{\text{V}}$_*E*_−$\dot{\text{V}}$CO_2_ relationship under the *Free* condition was used as the reference (1.0), the slope under *SL*_*fix*_ was steeper than that under *Free*, and the slope under *SF*_*fix*_ was lower than that under *Free*. These phenomena may be explained as follows: afferent feedback from the limb is important for locomotor-respiratory entrainment, whereby the discharge rhythm of sensory inputs can entrain a central respiratory pattern generator (Potts et al., 2005; Shevtsova et al., 2019; Le Gal et al., 2020).

### Locomotor-Respiratory Entrainment Irrespective of the Sinusoidal Change in Speed

In human studies, locomotor-respiratory entrainment has been observed when the locomotion speed is kept constant during walking (Bramble and Carrier, 1983; Bernasconi et al., 1995; McDermott et al., 2003; O’Halloran et al., 2012). Our locomotion protocol used the sinusoidal change in speed between 50 and 100 m⋅min^–1^. We thus considered the following possibilities: locomotor-respiratory entrainment forcing the synchronization of step movement and breathing rhythms is more likely to occur when the sinusoidal change in speed is synchronized with the sinusoidal change in SF (i.e., the *SL*_*fix*_ condition). According to our hypothesis, the *SF*_*fix*_ condition provided an unchanged B*f* (i.e., <1.0 breath⋅min^–1^), with the *Mx* for the B*f* approximately 24 breaths⋅min^–1^ at all periods (Supplementary Table 1 and Figure 4). Thus, the *Amp* of $\dot{\text{V}}$_*E*_ depended mostly on that for VT, which was characterized as the faster phasic response under the *SF*_*fix*_ condition (Figure 5G).

Apparently, the constrained B*f* under the *SF*_*fix*_ condition induced smaller *Amp*s of $\dot{\text{V}}$_*E*_ and P_*ET*_CO_2_ (Figures 5A,B,D), despite the absence of differences in the *Mx* for $\dot{\text{V}}$_*E*_ and P_*ET*_CO_2_ between the *SF*_*fix*_ and *SL*_*fix*_ conditions (Supplementary Table 1). Therefore, respiratory entrainment might be achieved by the same exercise hyperpnea without hypoventilation and lower P_*ET*_CO_2_ even under *SF*_*fix*_. These phenomena suggest the physiological significance of afferent feedback from the hindlimb locomotor generators for locomotor-respiratory coupling, whereby the discharge rhythm of sensory inputs can entrain a central respiratory pattern generator (Potts et al., 2005; Shevtsova et al., 2019; Le Gal et al., 2020). It was also reported that respiratory entrainment can be achieved by lumbar and/or cervical proprioceptive input stimulations in an isolated rat or mice brain stem-spinal cord preparation (Morin and Viala, 2002; Giraudin et al., 2012; Le Gal et al., 2020). Even though the *Amp* of the SL under the *SF*_*fix*_ condition was significantly (two times) larger than that under the control *Free* condition in all of the periods used herein, the *SF*_*fix*_ locomotion induced the sluggish dynamics of the *Amp* of $\dot{\text{V}}$_*E*_, $\dot{\text{V}}$O_2_, and $\dot{\text{V}}$CO_2_. Thus, the contribution of sinusoidal variation in SL to the adjustment in $\dot{\text{V}}$_*E*_ would be less than that in a *Free* condition.

### The Physiological Implications of Sinusoidal Cadence for Ventilation

Several research groups have compared the PS of $\dot{\text{V}}$_*E*_ dynamics under different experimental conditions during leg cycling, namely, between sinusoidal cadence with a constant pedal force and sinusoidal pedal force with a constant pedal cadence (Casaburi et al., 1978; Duffin, 2014; Caterini et al., 2016), and they observed that the PS of $\dot{\text{V}}$_*E*_ dynamics was much less for the cadence variation than for the sinusoidal pedal forcing variation. These studies used the fundamental concept that changing the frequency of limb movement during exercise would affect ventilation.

In contrast to those findings, our present investigation demonstrated that the *SL*_*fix*_ condition induced a significantly larger *Amp* of $\dot{\text{V}}$_*E*_ with similar *PS* and *Mx* values compared with the *SF*_*fix*_ condition (Figures 4, 5A and Supplementary Table 1). The larger *Amp* of $\dot{\text{V}}$_*E*_ was equal to that in the *Free* condition, even though the *Amp* of B*f* and VT tended to be larger under *SL*_*fix*_ compared with *Free* (Figures 5B,C). The smaller *PS* values for B*f* and the larger *PS* value for VT were specifically characterized under the *SL*_*fix*_ condition. The increased muscle contraction of sinusoidal cadence may thus be competitively related to the faster ventilatory drive to breathe (Takano, 1988; Caterini et al., 2016; Girardi et al., 2021). Notably, this specific adaptation was to the PS rather than the magnitude (Casaburi et al., 1977; 1978; Wells et al., 2007).

In this study, the phasic responses of $\dot{\text{V}}$_*E*_, B*f*, and P_*ET*_CO_2_ were not significantly different among the three locomotion patterns. The discrepancies between our results and those of the studies reported by Casaburi et al. (1978), Duffin (2014), and Caterini et al. (2016) may be explained by the different study settings (e.g., leg cycling vs. walking, and/or the mode of sinusoidal changes).

We also observed that the *Amp* of $\dot{\text{V}}$_*E*_ under the *SL*_*fix*_ condition was similar to that under the *Free* condition, even though the *Amp* of SF was sinusoidally altered (two times larger) compared with the *Free* condition (Figure 3A). Irrespective of the larger variation in SF during sinusoidal locomotion, the sinusoidal cadence under the present *SL*_*fix*_ condition inherently seemed to play a dominant role in the Amp responses of the ventilatory variables.

Considering the physiological mechanisms underlying these observations, it has been shown that the groups III and IV afferents in exercising limbs are stimulated by muscle contraction (Kaufman et al., 1983; Mense and Stahnke, 1983; Bruce et al., 2019). In this study, a greater *Mx* of $\dot{\text{V}}$_*E*_ under the *SL*_*fix*_ condition was manifested compared with the *Free* condition, which supports the concept that the limb movement frequency is a significant factor in the greater increase of $\dot{\text{V}}$_*E*_ during sinusoidal locomotion (Eldridge et al., 1981; Eldridge, 1994).

### Influence on $\dot{\text{V}}$_*E*_-$\dot{\text{V}}$CO_2_ Linkage During Three Locomotion Patterns

When we used the slope of the $\dot{\text{V}}$_*E*_-$\dot{\text{V}}$CO_2_ relationship under the *Free* condition as the reference (1.0), the slope under *SL*_*fix*_ (1.2) was steeper than that under *Free*, and the slope under *SF*_*fix*_ was gentler than that in *Free* (Figure 7). The gait pattern for a sinusoidal variation of the SF may cause greater exercise hyperpnea, which can be interpreted as an overreaction of the additional stimulation on the respiratory center *via* the supraspinal locomotor center (DiMarco et al., 1983; Fukuoka et al., 2017). In other words, the greater $\dot{\text{V}}$_*E*_ at a given $\dot{\text{V}}$CO_2_ under *SL*_*fix*_ locomotion suggests that the central feed-forward command (Bell, 2006) or upward information from the afferent neural activity was stimulated by the sinusoidal locomotive cadence. Therefore, these neural drives could be partly related to the steeper slope of the $\dot{\text{V}}$_*E*_−$\dot{\text{V}}$CO_2_ relationship under *SL*_*fix*_ rather than the humoral outcome *via* the equivalent metabolic demand under *Free* (Eldridge and Waldrop, 1991; Waldrop et al., 1996; Forster et al., 2012; *Fukuoka et al., 2017*).

Considering *Free* locomotion, our subjects’ preferred SL was likely to have a lower metabolic demand (Supplementary Table 2), and a significant main effect of the locomotion patterns was observed on the *Mx* for $\dot{\text{V}}$_*E*_, and there was a significant difference between the *Free* and *SL*_*fix*_ conditions in the 5-min period (*p* = 0.015, Supplementary Table 1). Thus, the steeper slope of the $\dot{\text{V}}$_*E*_−$\dot{\text{V}}$CO_2_ relationship under *SL*_*fix*_ might be attributed to a greater *Mx* for $\dot{\text{V}}$_*E*_. The observed differences in the ventilatory response between our *SL*_*fix*_ and *Free* conditions would be attributed to the larger *Amp* of SF, which contributed to the neuromuscular afferent flow into the medial brain and respiratory-locomotor generation center (Eldridge et al., 1981, 1982; Eldridge, 1994).

In contrast, the slope under *SF*_*fix*_ (0.7) was lower than that under *Free* even though the *Mx* of $\dot{\text{V}}$_*E*_, $\dot{\text{V}}$O_2_, and $\dot{\text{V}}$CO_2_ were not significantly different between the *SL*_*fix*_ and *SF*_*fix*_ conditions (Figure 7 and Supplementary Table 2). The remarkable difference in the slope of the $\dot{\text{V}}$_*E*_−$\dot{\text{V}}$CO_2_ relationship was due to the *Amp* of the SF. The lower slope was caused by the depressed $\dot{\text{V}}$_*E*_, which we attribute to the entrained breath frequency under *SF*_*fix*_. The neural stimulus related to locomotion appeared to interact with the chemical stimulus in a predominantly “additive” manner (DiMarco et al., 1983). This finding may support our hypothesis that the modification of respiratory changes occurs indirectly *via* the gait pattern generator by maintaining the constrained SF (Le Gal et al., 2020).

We speculate that the very close association between $\dot{\text{V}}$_*E*_ and $\dot{\text{V}}$CO_2_ at all locomotion patterns (*r* = 0.83−0.92, Figure 7) provides a rationale for considering mechanisms unrelated to the motor act (such as humoral factors) to explain the link between $\dot{\text{V}}$_*E*_ and $\dot{\text{V}}$CO_2_ (Whipp et al., 1982). A major question in the physiological interpretation of the strong linkage between $\dot{\text{V}}$_*E*_ and $\dot{\text{V}}$CO_2_ is that the CO_2_ amount is an adjustment factor, but the chemoreceptor does not sense the CO_2_ amount and respond to the partial pressure of CO_2_ (Forster et al., 2012). Considering that chemoreceptors are always involved, the alteration of P_*ET*_CO_2_ could be involved as a derivative signal to the arterial and central chemoreceptors, which are further related to $\dot{\text{V}}$_*E*_ (Forster et al., 2012; Ebine et al., 2018).

It has been recognized that a sinusoidal exercise protocol would indicate a lesser contribution to the ventilatory changes from the neural signals through the motor activity and/or central command (Forster et al., 2012). Wells et al. (2007) chose the shorter period of 1 or 2 min of sinusoidal change in walking speed between 3.2 and 6.4 km⋅h^–1^ (approximately 53 and 107 m⋅min^–1^) to emphasize the faster $\dot{\text{V}}$_*E*_ response against locomotion. Contrary to their observations, the *Amp* values of $\dot{\text{V}}$_*E*_ in this study were tightly coupled to those of $\dot{\text{V}}$CO_2_ during sinusoidal walking. The contribution of limb movement to exercise hyperpnea has thus been a matter of debate (Casaburi et al., 1978; Duffin, 2014; Ward, 2019). Moreover, exercise training can affect exercise hyperpnea through attenuation of a neural drive that is either feedforward or feedback in nature (Miyamoto et al., 2012).

### Limitations

We set up three locomotive patterns based on the assumption of a sine wave speed, and thus we did not experiment with changing the SF sinusoidally at a fixed load (in this case, speed), which has been treated in leg cycling (Casaburi et al., 1978; Wells et al., 2007). Further studies using sinusoidal changing of the SF may be necessary.

It remains difficult to determine the relative contributions of the mechanoreflex vs. the metaboreflex to ventilatory control in humans (Olson et al., 2010, 2014). The novel findings of our present investigation possibly demonstrate that the afferent feedback from skeletal muscle triggers a marked increase in the slope of the $\dot{\text{V}}$_*E*_−$\dot{\text{V}}$CO_2_ relationship under *SL*_*fix*_ locomotion. However, we were unable to differentiate the involvements of peripheral afferent feedback from central command without the direct measures. The individual contribution of both neural factors to the ventilatory response in humans cannot be evidently stated from the experimental results in this study.

## Conclusion

In summary, the *SL*_*fix*_ locomotion pattern enlarged the *Amp* of $\dot{\text{V}}$_*E*_ and metabolic responses ($\dot{\text{V}}$O_2_ and $\dot{\text{V}}$CO_2_, and HR) compared with the *SF*_*fix*_ pattern (i.e., 120 steps⋅min^–1^). Moreover, the *Amp* of *Bf* remained unchanged (<1.0 beats/min) under *SF*_*fix*_. The slope of the $\dot{\text{V}}$_*E*_−$\dot{\text{V}}$CO_2_ relationship was steeper by 1.23 times under *SL*_*fix*_ and was gentler by 0.72 times under *SF*_*fix*_ when the slope under the control (*Free*) condition was used as the reference. These results are explained as follows: afferent feedback from the limb is important in locomotor-respiratory entrainment, whereby the discharge rhythm of sensory inputs can entrain central respiratory-pattern generation. The PSs of $\dot{\text{V}}$_*E*_, $\dot{\text{V}}$O_2_, and $\dot{\text{V}}$CO_2_ responses were unaffected at any of the locomotion patterns. Such sinusoidal wave manipulation of locomotion variables may offer new insights into the dynamics of exercise hyperpnea.

## Data Availability Statement

The raw data supporting the conclusions of this article will be made available by the authors, without undue reservation.

## Ethics Statement

The studies involving human participants were reviewed and approved by the Ethics Committees of the Institutional Review Board of Doshisha University (no. 1045). The patients/participants provided their written informed consent to participate in this study.

## Author Contributions

MF, TA, and YF conceived and design of the study. MF, TA, and KK collected the data. MF, MH, KK, and YF interpreted of the data. MF, MH, and YF wrote, reviewed, and approved the final manuscript. All authors contributed to the collection of data.

## Conflict of Interest

The authors declare that the research was conducted in the absence of any commercial or financial relationships that could be construed as a potential conflict of interest.

## Publisher’s Note

All claims expressed in this article are solely those of the authors and do not necessarily represent those of their affiliated organizations, or those of the publisher, the editors and the reviewers. Any product that may be evaluated in this article, or claim that may be made by its manufacturer, is not guaranteed or endorsed by the publisher.
