# Supplementary figures and images for: Influence of Step Frequency on the Dynamic Characteristics of Ventilation and Gas Exchange During Sinusoidal Walking in humans
Source: Front Physiol. 2022 Apr 12;13:820666. doi: 10.3389/fphys.2022.820666 (PMC9039249; doi:10.3389/fphys.2022.820666)

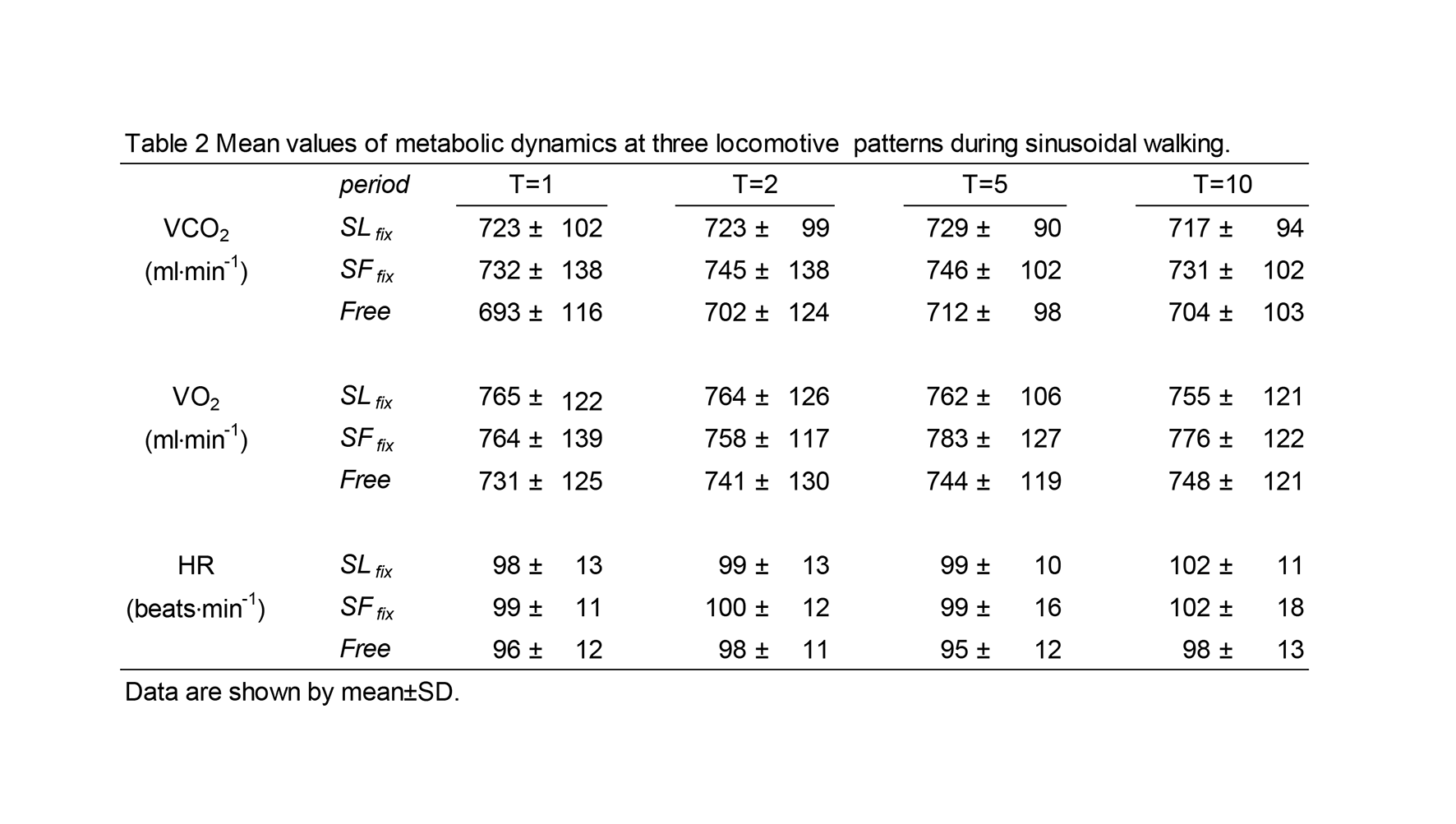

Supplement: Supplementary file 1 [file Image_1.TIF]

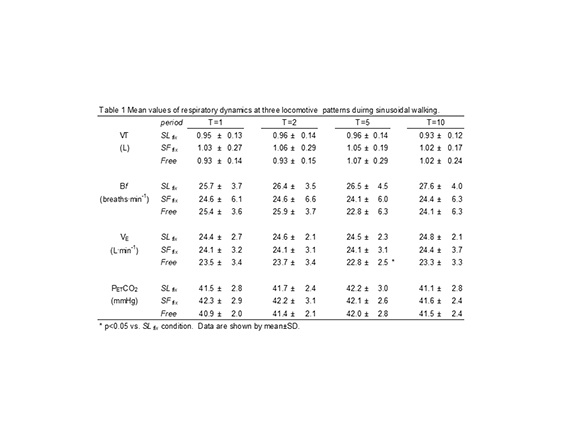

Supplement: Supplementary file 2 [file Image_2.TIF]

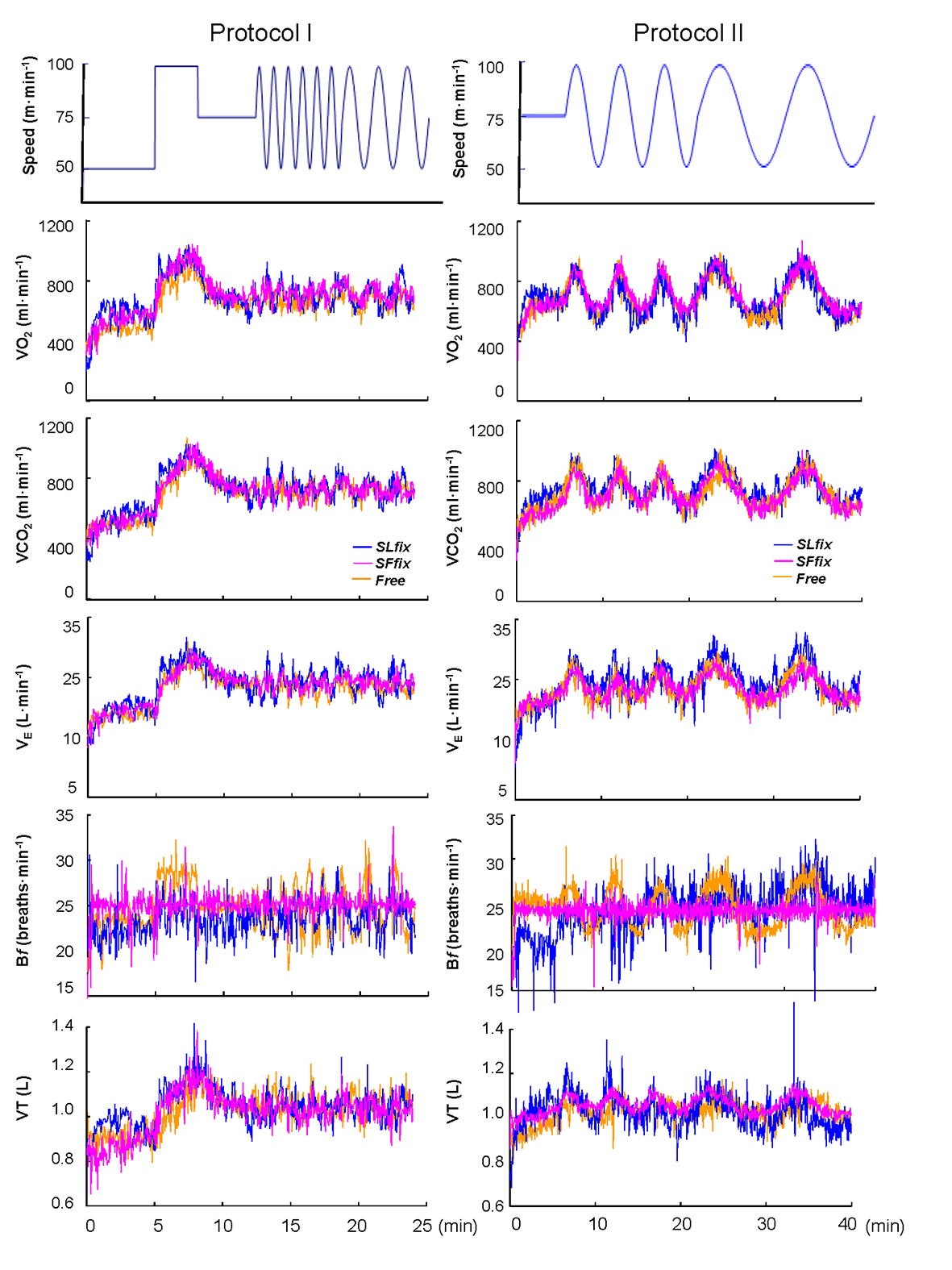

Supplement: Supplementary file 3 [file Image_3.TIF]
